# Physical Activity as a Treatment for Social Anxiety in Clinical and Non-clinical Populations: A Systematic Review and Three Meta-Analyses for Different Study Designs

**DOI:** 10.3389/fnhum.2021.653108

**Published:** 2021-06-11

**Authors:** Maya A. Zika, Linda Becker

**Affiliations:** Department of Psychology, Friedrich-Alexander University Erlangen-Nürnberg, Erlangen, Germany

**Keywords:** social anxiety, social-anxiety disorder, social phobia, fear of negative evaluation, physical activity, physical exercise, meta-analysis

## Abstract

The fear of being in the focus of attention in social situations can develop into a social anxiety disorder (SAD). The classical treatment for SAD is cognitive behavioral therapy, which is in many cases accompanied by drug treatments. A promising alternative treatment is physical activity (PA) interventions, because regular PA has been shown to be suitable for reducing anxiety in general. We conducted a pre-registered systematic review and meta-analysis (PROSPERO registration no. CRD42020191181) as well as two additional searches. Our aim was to investigate whether PA interventions are a suitable treatment for SAD and whether PA is suitable for reducing social anxiety (SA) in general. For studies with randomized controlled trial designs, a not statistically significant effect of medium size toward lower general SA symptomatology was found in the PA group in comparison with the control group (*d* = −0.24, *p* = 0.377). For studies with longitudinal designs, significantly lower SA symptoms were found after PA treatments (*d* = −0.22, *p* = 0.001). The effect of PA on SA was stronger for adults than for children and adolescents (*p* = 0.003). For cross-sectional studies, a small negative association between SA symptoms and the amount of PA was found, i.e., lower SA was found for people who were more physically active (*r* = −0.12, *p* = 0.003). We conclude that PA is a promising means for the (additional) treatment of SAD or to reduce SA in general in non-clinical samples, but more research in which high-quality studies with randomized controlled trial designs are used is needed. Furthermore, open questions with respect to moderating variables (e.g., age, sex, BMI, type of intervention, stress, amount of regular PA before the intervention, and comorbidities) remain still open.

## Introduction

### Rationale

The fear of being in the focus of attention in social situations and of being embarrassed can develop into a clinically relevant mental disorder. This social phobia or social anxiety disorder (SAD; DSM-V 300.23, ICD-10: F40.1) is one of the most common anxiety disorders worldwide (Bandelow and Michaelis, 2015; Stein et al., 2017) with a lifetime prevalence of 1.8% (Mohammadi et al., 2020) and a point prevalence of 4.4% (Camuri et al., 2014). Social anxiety disorder can be found in all age groups (Ohayon and Schatzberg, 2010; Karlsson et al., 2016; Grenier et al., 2019; Mohammadi et al., 2020) and often has its onset in childhood (Otto et al., 2001). Furthermore, prevalence increases in adolescence (Wright et al., 2020). Social anxiety disorder often occurs comorbidly with other psychiatric disorders, such as depression and substance dependency (Ruscio et al., 2008; Ohayon and Schatzberg, 2010; Stein et al., 2017; Prior et al., 2019; Rozen and Aderka, 2020). One of the core symptoms of SAD is fear of negative evaluation (FNE; Hartmann et al., 2010). In most cases, people with SAD have problems to perform well in social situations (Wells et al., 1995) and have altered social cognitive abilities, such as dysregulated shyness (Nikolić, 2020) as well as selective attention and reporting bias for negative social stimuli (Amir et al., 2003; Garner et al., 2006; Bublatzky and Alpers, 2017). Furthermore, in many cases, people with SAD hold firm beliefs about the importance of making good impressions to others while believing that they come across badly (Leary, 2001). Social anxiety (SA) can be perceived as extremely stressful and is often accompanied by avoidance behavior or facing social situations with intense fear. This fear and other SAD symptoms are often accompanied by physiological arousal (e.g., blushing, sweating, trembling, fast heart rate, upset stomach, nausea, or muscle tension; Kessler et al., 1999; Rösler et al., 2021), which are related to physiological and psychological stress.

Particularly, the avoidance behavior—as another core symptom of SAD—makes it challenging to find suitable therapeutic treatments. If help is sought, the most widely used and most effective treatment approach is cognitive behavioral therapy (CBT), which is in many cases accompanied by drug treatment of the anxiety symptoms (Gould et al., 1997; Rowa and Antony, 2005; Pontoski et al., 2010). However, a recent meta-analysis has shown that CBT in adolescents with SAD is less effective than for other anxiety disorders (Evans et al., 2020), which highlights the importance of finding alternatives or treatments that could complement CBT. A common alternative for the treatment of anxiety disorders in general is the combination of CBT with physical activity (PA) treatments with the goal to further reduce anxiety symptoms and to improve physical besides mental health (Woll and Bös, 2004; Penedo and Dahn, 2005).

Regular PA can lead to an improvement in well-being, life satisfaction, and cognitive functioning and is associated with positive effects on mood and anxiety (Netz et al., 2005; Sharma et al., 2006; Warburton et al., 2006). In contrast, physical inactivity has been shown to be associated with increased symptoms of depression and anxiety (Aichberger et al., 2010). The anxiolytic effects of PA have been reported in various studies and meta-analyses (Petruzzello et al., 1991; Ströhle, 2009; Conn, 2010; Rebar et al., 2015). Several mechanisms may explain these anxiolytic effects of PA. One approach is the thermogenic hypothesis that assumes that body temperature is elevated during exercising, which is detected by the brain and which triggers physiological processes that lead to a reduction of anxiety symptoms (Lim et al., 2008). Another explanation is the time-out hypothesis, which suggests that being active provides a distraction from daily life routines and stressors, which, therefore, leads to a reduction of anxiety symptoms (Ströhle et al., 2009). Another approach is the mastery hypothesis, which states that success achieved during PA leads to a decrease of negative thoughts and feelings and, thus, to a reduction of anxiety symptoms (Asmundson et al., 2013). Furthermore, it has been found that PA leads to a reduction of anxiety sensitivity (LeBouthillier and Asmundson, 2015). Moreover, the biological mechanisms underlying the anxiolytic effects of PA have been discussed. These include changes in monoamine levels and activity of the hypothalamic–pituitary–adrenal axis and upregulation of neurotrophic growth factors, as well as changes in neuronal structures, such as the limbic system (see Wegner et al., 2014 for a comprehensive overview). Overall, PA has the potential to be a promising means for reducing anxiety, and thus, the treatment of SAD and several models and mechanisms to explain these effects have been suggested.

However, one hurdle that must be overcome to make PA indeed a suitable treatment is the avoidance behavior, which is often found in SAD. One solution could be engaging in PA alone without the presence of others (i.e., doing individual sports instead of team sports). On the other hand, if the fear is overcome and people with SAD participated in team sports, this could have an additional therapeutic benefit, because positive social relationships can have a positive impact on psychological stress and SA (Eime et al., 2013). In general, little is known about differences in treatment efficacy between participating in team sports and individual sports with respect to anxiety disorders. Most previous studies focused on anxiety disorders in general and did not report subanalyses for SAD and report cross-sectional data (e.g., Pasco et al., 2011; Faulkner and Tamminen, 2016). Longitudinal findings remain limited and mixed (Agans and Geldhof, 2012; Nixdorf et al., 2016).

To summarize, it is well-known that PA can have beneficial effects on physical and mental health and that PA can reduce anxiety (e.g., Landers and Petruzzello, 1994). However, little is known whether PA is also suitable for the treatment of SAD and the underlying mechanisms are yet not fully understood. Furthermore, whether team or individual sports are more suitable for the treatment of SAD remains an open question.

### Objectives

Our main review questions were whether PA interventions are suitable treatments for SAD and whether PA is suitable for reducing SA in general. Furthermore, we aimed to investigate which treatment form (e.g., group vs. individual, endurance vs. resistance training, with or without the presence of a therapist/trainer) is more effective. Further review questions were if the reduction of stress-associated symptoms is a moderator of treatment efficacy and whether treatment efficacy is associated with patient characteristics (e.g., age, sex, general health, education, and comorbidities). Three independent searches and analyses for different study designs were conducted. First, we focused on randomized controlled trials (RCTs). Second, we also searched for longitudinal studies, and third, we included cross-sectional studies. In all analyses, we differentiated between clinical (i.e., with SAD diagnosis) and non-clinical populations.

## Part 1: Randomized Controlled Trials

### Introduction

In the first part of our study, high-quality studies, in which randomized controlled trial designs (RCT) were used, were considered only. The aim was to compare treatment efficacy of PA interventions to control interventions (either a passive control group or a non-PA intervention) for people with SAD diagnosis.

### Materials and Methods

#### Protocol and Registration

This systematic review and meta-analysis has been registered with PROSPERO (CRD42020191181) in June 2020 before starting with the literature search. The reporting of the review is consistent with the Preferred Reporting Items of Systematic Reviews and Meta-Analyses (PRISMA; Moher et al., 2009) guidelines.

#### Search Strategy and Databases

The search strategy was developed through discussions between the authors. The following electronic bibliographic databases were searched for peer-reviewed articles in English language only: APA PsycArticles, APA PsycINFO, PubMed/MEDLINE, SPORTDiscus, Sports Medicine & Education Index, Psychology and Behavioral Sciences Collection, and Web of Science. Search terms were divided into three blocks and were adjusted according to the respective databases' Thesaurus and Medical Subject Headings (MeSH) terms. The first block included search terms related to SAD (e.g., social anxiety, social phobia, performance anxiety), the second included terms that are related to PA (e.g., physical activity, exercise, sport, fitness, walking, dancing), and the third block included terms that are related to interventions (e.g., treatment or RCT). The full list of search terms is provided in Supplementary Material 1. Reference lists from retrieved full-text articles were also searched for additional studies. Unpublished studies, conference proceedings, and other gray literature were not considered. There was no limitation for the year of publication.

#### Article Selection and Data Extraction

After removal of duplicates, a two-stage screening approach was performed. First, titles and abstracts of retrieved studies were screened independently by the two review authors (MZ and LB) in order, to identify articles that met the inclusion criteria. For this first screening, the online screening tool Rayyan (Ouzzani et al., 2016) was used. Second, full texts were independently assessed by the two reviewers for eligibility and were afterwards proceeded to data extraction. Disagreements between the ratings were resolved through discussion until a consensus was reached. One of the initial inclusion criteria was that we intended to include samples with SAD diagnosis only. Healthy populations and people with other anxiety disorders without a comorbid SAD were excluded. Furthermore, RCTs were considered as study design only. Interventions must have been any form of an exercise intervention (e.g., endurance exercise, resistance training, gymnastics, dancing, hiking, or walking). Relaxation trainings (e.g., progressive muscle relaxation, autogenic training, yoga, Pilates, tai-chi, qi-gon), mindfulness-based trainings, cognitive trainings, and psychological interventions (e.g., CBT) were excluded. Furthermore, the studies must include either a passive (e.g., waiting control group) or active control group (e.g., CBT, cognitive training, general health education, or a relaxation training). Because four studies were found only which met the original inclusion criteria (i.e., an RCT design and a clinical SAD sample), we decided to additionally include non-clinical samples.

A standardized, pre-piloted spreadsheet was used to extract data from the final selected studies. It included study identification (i.e., authors, setting, country, journal), sample characteristics [age, sex, body mass index (BMI), ethnicity, education, diagnosis, and comorbidities], methodological specifications, details of the intervention or assessment of PA, assessment of the outcome (SA measure), results (e.g., means and standard deviations, *p*-values, and effect sizes), suggested covariates, study limitations, additional information (e.g., key conclusions, funding source, potential conflicts of interest), and decision on study inclusion or exclusion. If necessary, missing data were requested from the study's corresponding author *via* email. The authors of two studies (Rocha et al., 2014; Zink et al., 2019) were contacted, and all responded to the request. Furthermore, a participants–interventions–comparisons–outcomes–study design (PICOS) table was created, which is provided as Supplementary Material 2.

#### Risk-of-Bias (Quality) Assessment

All selected studies were evaluated concerning their methodological quality. Both review authors (MZ, LB) rated the risk-of-bias (ROB) independently by using the “Quality Assessment Tool of Controlled Intervention Studies” from the National Heart, Lung, and Blood Institute (2019). Disagreements in the rankings were resolved through discussions. The scale rates of ROB were good, fair, and poor ROB depending upon the information provided in the studies. High ROB translates to a rating of poor quality. Low ROB translates to a rating of good quality. For ROB visualization, the web tool by McGuinness and Higgins (2020) was used.

#### Statistical Data Analysis

Cohen's *d* was considered as measure for effect size for the meta-analysis. If not reported, Cohen's *d* was calculated according to the following formula:

$$\begin{array}{l} \;d\left(RCT\right)=\;\frac{M\left(treatment\right)-M\left(control\right)}{\sigma }\\ \text{with }\sigma =\sqrt{\frac{\left[n\left(treatment\right)-1\right]\times {SD\left(treatment\right)}^{2}+\left[n\left(control\right)-1\right]\times {SD\left(control\right)}^{2}}{\left[n\left(treatment\right)+n\left(control\right)-2\;\right]}} \end{array}$$

for comparison between the strength of anxiety symptoms between the treatment and the control group after the intervention. Because of missing data, it was not possible to compare the mean changes (baseline and after intervention) in SA between the groups. We considered *d* < 0.2 as small, between 0.2 and 0.5 as medium, and >0.5 as strong effects (Fritz et al., 2012).

For the meta-analyses, the tool meta-essentials (Suurmond et al., 2017) was used. Forest plots were used for the visualization of effect sizes. The main outcome was general SA symptomatology. If several measures were reported, mean outcome measures were calculated. Furthermore, we planned to conduct subanalyses for different core symptoms of SAD as outcome variable to investigate whether they are differentially related with PA interventions. Due to the number of search results, this was only possible for FNE. To consider study quality, we planned to conduct the analyses for all eligible studies first and, additionally, for the fair rated studies separately.

We aimed to conduct moderator analyses for the moderator variables age, percentage of female participants, BMI, comorbidities, type of PA (e.g., endurance vs. resistance or individual vs. team sports), and stress if enough data were available. Furthermore, we aimed to conduct further subgroup analyses to compare the effects for healthy and clinical populations. An adjusted α-level of α_adjusted_ = 0.05/7 = 0.0071 was used because seven separate analyses were planned (for age, sex, BMI, comorbidities, stress, type of PA, and clinical vs. non-clinical). Random effects models were used for all analyses. Cochrane's *Q* was considered as a measure for heterogeneity of variance.

### Results

#### Overview of Search Results

**Figure 1:**
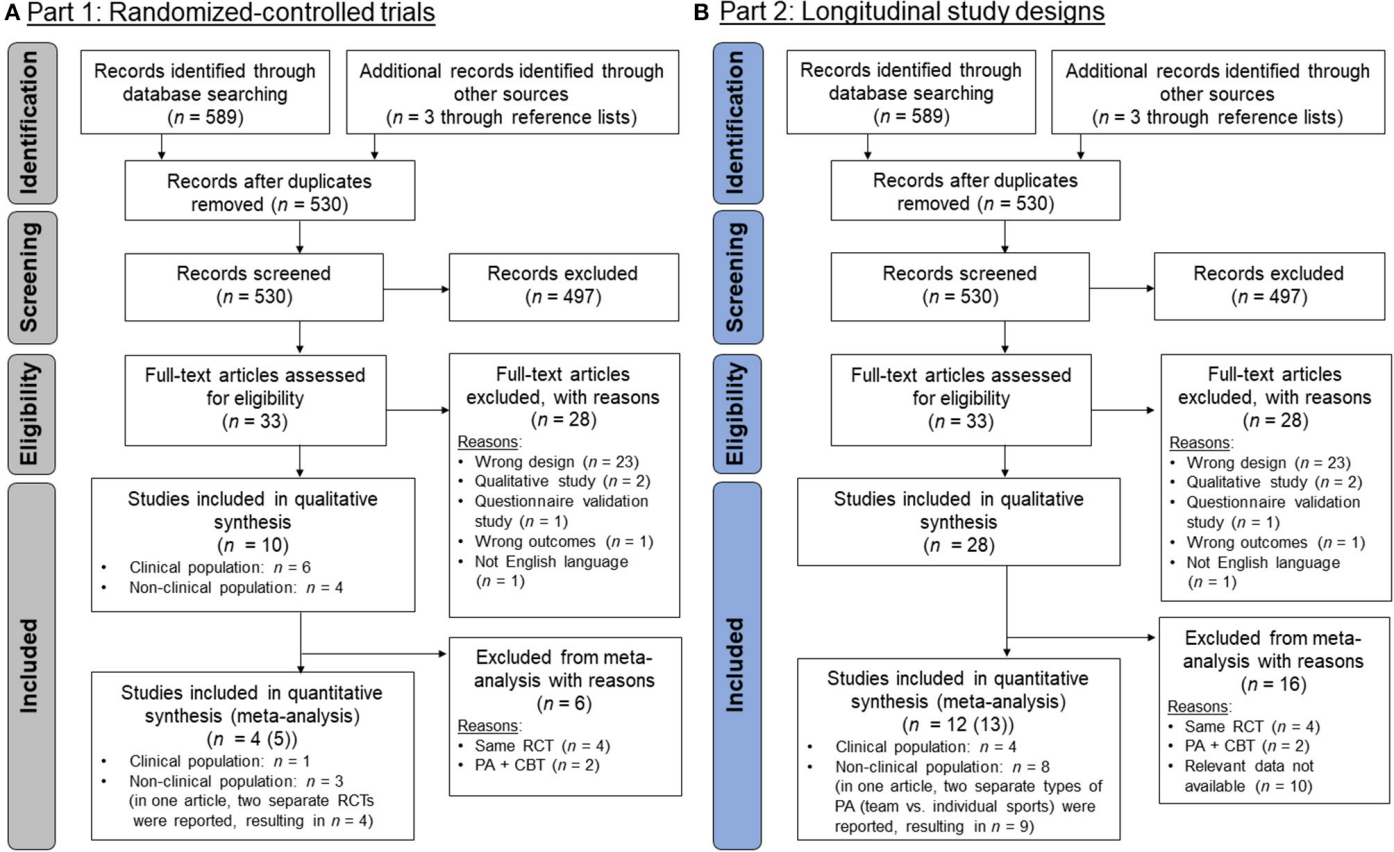
PRISMA flow-chart diagrams (Moher et al., 2009): **(A)** Studies with a randomized-controlled trial (RCT) design (part 1); **(B)** studies with a longitudinal design (part 2). The RCTs that were found in **(A)** were also included in **(B)**. The steps until inclusion were the same part 1 and 2 until the screening process and, therefore, the numbers are equal.

With the original search strategy, four studies were found which were included in the qualitative synthesis (i.e., the review). From these four studies, one was eligible for inclusion in the meta-analysis. When additionally including non-clinical populations, six additional studies with an RCT design were found, from which four (three articles, one was included twice, because two separate RCTs were reported) were included in the meta-analysis. The whole PRISMA flowchart diagram (Moher et al., 2009) is provided in Figure 1A.

Reasons for exclusion from quantitative synthesis were that in two cases (one clinical and one non-clinical), the same RCTs were described in numerous articles (including the same participants, outcome measures, and interventions, but which were focusing on slightly different research questions). We decided to include only one of each group of studies. We considered the study of Jazaieri et al. (2012) to be representative for Jazaieri et al. (2012, 2016) and Goldin et al. (2012, 2013). Furthermore, the article by Merom et al. (2008) was considered as representative for Merom et al. (2008) and Phongsavan et al. (2008). However, this study (Merom et al., 2008) had nevertheless to be excluded, because a combined intervention (PA + CBT) was reported, and it was not possible to separate the effect of PA from the effect of CBT. The study by Jelalian et al. (2011) was also excluded from the meta-analysis because of a combined PA + CBT intervention. The study by Lokös et al. (2013) was included twice into the meta-analysis, because two separate interventions (a swim training and a complex sport therapy) and separate control groups were reported in this study. An overview about the characteristics of all 10 RCTs is provided in Table 1.

**Table 1:** Characteristics of the randomized controlled trials.

**References**	**Population**	**Main diagnosis**	***N***	***N* post (*n*_**int**_; *n*_**con**_)**	**Dropout rate (%)**	**Mean age (SD)**	**Sex (% female)**	**Ethnicity**	**Education**/ **employment**	**SA** **assessment**	**FNE** **assessment**	**PA** **assessment**	**Intervention**	**Frequency PA intervention**	**Duration**/ **intensity single** **PA unit**	**Total duration** **PA intervention**	**Control group treatment**
Hartmann et al. (2010)^*^	NCL	NA	502	449 (*n*_int_ = 277, *n*_con_ = 172)	52	9.3 (2.2)	51	Swiss parents (63%), two foreign parents (23%), one Swiss parent and one foreign parent (14%)	Elementary school students	SASC-RD	FNE-scale of the SASC-R.D G. v.	NA (intervention)	Daily physical education class in school, aerobic exercise, PA homework, encouragement of activities during breaks	Daily	Physical education class (2 × 45 min) + several short activity breaks per day	One academic year	Usual school curriculum including three physical education lessons per week (45 min each)
Goldin et al. (2012, 2013), Jazaieri et al. (2012, 2016)^*^	CL	SAD	56	42 (*n*_int_ = 18; *n*_con_ = 24)	25	32.69 (8.4)	52	Caucasian (41%), Asian (45%), Hispanic (7%), multiracial (7%)	Education: 16–17 years	LSAS-SR; SIAS-S	NR	NA (intervention)	Aerobic exercise	Weekly	2 individual aerobic exercise sessions (duration NR) + one group AE session	2 months	MBSR
Jelalian et al. (2011)	NCL	NA	118	89 (*n*_int_ = 44, *n*_con_ = 45)	25	14.2 (0.93)	69	Caucasian (78.7%), African-American (12.4%), Hispanic (5.6%), other (3.4%)	High school students	SAS-A	NR	NA (intervention)	CBT + PA	Weekly	60 min aerobic exercise	16 weeks	CBT + PEAT
Lokös et al. (2013)^*^	NCL	NA	82	82 (*n*_int1_ = 26, *n*_con1_ = 26, *n*_int2_ = 15, *n*_con2_ = 15)	NR	9.85 (0.96)	54	NR	Elementary school students	STAI-C	SASC-N.E	NA	Swim training or complex sports therapy	Swim training: twice a week; complex sports therapy: two weekly swim sessions of 60 min each, one weekly 60-min session of indoor sports and one 60-min session of outdoor sports	Swim training: 60 min each session (2× /week); complex sports therapy: 60 min swim training (2× /week), 60 min indoor sports (1× /week), 60 min outdoor sports (1× /week)	18 months	No organized sport activities
Merom et al. (2008), Phongsavan et al. (2008)	CL	Fulfillment of DSM-IV criteria for primary diagnosis of GAD, PD, or SP	85	41 (*n*_int_ = 21, *n*_con_ = 20)	45	39 (12.1)	76	NR	49% being in a full- or part-time employment (intervention group)	DASS-21	NR	Self-report PA measures derived from the Active Australian Questionnaire protocol Australian Institute of Health Welfare., 2003, pedometers	CBT + PA	Daily	Individual; gradual increase in the number of 30-min sessions of moderate intensity exercise to achieve at least 150 min/week	10 weeks	General CBT + ED
Yu et al. (2020)^*^	NCL	NA	203	171 (*n*_int_ = 99, *n*_con_ = 72)	16	9.8 (0.7)	20.5	Asian	Elementary school students	SAS-C	NR	NA (intervention)	School-based nutrition education and PA	Daily on school days + PA education in class every 2 months	20 min jogging + one extra 40-min gym class (rope skipping, badminton, and 200-m relay race) + PA educational class 4 × 60 min	8 months	Usual practice with no intervention

*CBT, cognitive behavioral therapy; CBT + ED, CBT and additional education; CBT + PA, CBT and additional exercise therapy; CBT + PEAT, CBT and additional peer-enhanced adventure therapy; CL, clinical; FNE, fear of negative evaluation; FNE-scale of the SASC-R.D G. v., the Fear of Negative Evaluation scale (FNE-scale) from the German version of the SASC-R.D (Melfsen and Florin, 1997); DASS-21, Depression and Anxiety Stress Scale (Lovibond and Lovibond, 1995); GAD, generalized anxiety disorder; LSAS-SR, Liebowitz Social Anxiety Scale—Self-Report (Liebowitz et al., 1985; Fresco et al., 2001); MBSR, mindfulness-based stress reduction; N, sample size; n_con_, sample size in the control group; n_int_, sample size in the intervention group; NA, not applicable; NCL, non-clinical; NR, not reported; PA, physical activity; PD, panic disorder; SA, social anxiety; SAD, social anxiety disorder; SAS-A, the Social Anxiety Scale for Adolescents (La Greca and Lopez, 1998), originally adapted from the Social Anxiety Scale for Children—Revised (La Greca and Stone, 1993); SAS-C, Social Anxiety Scale for Children (SASC; Sanna et al., 2009); SASC-N.E, Fear of Negative Evaluation scale of the Social Anxiety Scale for Children (SASC-H, Sipos and Rákos, 1991); SASC-RD: The Social Anxiety Scale for Children–Revised (La Greca and Stone, 1993); SIAS-S, The Social Interaction Anxiety Scale (Rodebaugh et al., 2007); SD, standard deviation; SP, social phobia; STAI-C, State Trait Anxiety Inventory for Children (Spielberger and Edwards, 1973), STAI-C adapted for Hungarian children (Sipos and Sipos, 1979; Sipos et al., 1985)*.

* *Included in the meta-analysis*.

#### Qualitative Synthesis (Systematic Review)

##### Participants

The total number of subjects in the six studies (out of the 10 studies that were considered as eligible, from which the same RCTs were reported in four cases) that were included in the qualitative synthesis was 1,046 with a median of 101.5 per study and a range of 56 to 502. The studies were conducted between 2010 and 2020. In two of the studies (Merom et al., 2008; Jazaieri et al., 2012), a clinical population (with SAD diagnosis) was described, and in four studies, non-clinical populations were described. The mean age of the study participants was 19 years (*SD* = 12.1). Age ranged from 9 to 39 years; 53.8% of the population were female (range from 20.5 to 76%). In one RCT (Merom et al., 2008), the participants suffered from an addiction disorder, and in another study (Lokös et al., 2013), participants had functional spinal column disorders (FSCD) and/or asthma. Participants had no psychiatric or somatic comorbidities in the other RCTs. Ethnicity of the participants was Caucasian in most cases, followed by Asian and Hispanic. The amount of regular PA before the intervention was reported by Merom et al. (2008) only. In this study, 36% of the participants in the intervention group were inactive (<30 min/week), 18% were moderately active (30–149 min/week), and 21% were active (≥150 min/week). Furthermore, Jazaieri et al. (2012) reported that they excluded participants who exercised regularly, which was defined as three or more times a week for more than 2 months.

##### Types of Intervention and Associations Between Treatment and Social Anxiety Symptomatology

The only study in which an RCT design was used and a PA intervention was conducted in a clinical sample diagnosed with SAD was Jazaieri et al. (2012). To fit our research question, we considered the group that was initially assigned as the control group as the interventional group. The aim of the original study was to compare a mindfulness-based stress reduction (MBSR) program with a PA intervention as well as with an untreated SAD control group. The PA intervention was a 2-month aerobic intervention which included a gym membership. Participants were required to complete at least two individual aerobic sessions at moderate intensity and one group aerobic session per week. The PA intervention was more efficaciously in lowering SAD symptomatology than in the untreated SAD control group. Both PA and MBSR reduced SAD symptomatology and there was no significant difference between MBSR and PA in efficiency.

In the study by Hartmann et al. (2010), a daily physical education class in school was offered. Two additional lessons of 45 min each (given by physical education teachers), several short activity breaks per day during academic lessons, PA homework, and playgrounds adapted and improved to encourage activities during school breaks and before and after school were provided. In addition to aerobic exercise, the program included motor tasks, strength training, and play activities. The PA intervention did not reduce FNE (as the SA outcome measure) in this study.

In Yu et al. (2020), an 8-month school-based nutrition education and physical activity program was conducted. Parents were invited to participate in health education curriculums and doing exercises at home together with their children. Additionally, mandatory daily exercises on school days as well as lifestyle modifications were implemented. The program consisted of a 20-min class break in the morning in the form of jogging. One extra gym class of 40 min in the afternoon after school was provided, which included three forms of exercises (rope skipping, badminton, and 200-m relay race). The lifestyle modification was mainly performed through nutrition and PA education classes. The PA intervention showed a lowering effect in SA symptoms from baseline to post-intervention in the treatment group, which was stronger than in the control group.

Lokös et al. (2013) provided an 18-month swim training, which consisted of 2 weekly swimming sessions of 60 min in one intervention group. In another intervention group, complex sports therapy was provided for 18 months, which included 2 weekly swimming sessions of 60 min, 1 weekly 60-min session of indoor sports, and one 60-min session of outdoor sports. Both PA interventions showed a decrease in self-reported SA symptoms compared with matched control groups. Comparing the two interventions, the complex sports therapy showed stronger reduction of SA symptoms than the swim training.

In the study of Merom et al. (2008), 90-min exercise sessions were delivered once a week for 8 weeks for panic disorder and generalized anxiety disorder patients, or 10 weeks for patients diagnosed with SAD. The exercise intervention was provided additionally to general CBT. The program aimed to gradually increase the number of 30-min sessions of moderate-intensity exercises (e.g., brisk walking) at the participant's own choice of day, time, and environment. During the first three general CBT weeks, extended program meetings were provided to discuss the benefits of exercising, to provide general instructions, and to provide knowledge about the difference between moderate- and vigorous-intensity activities by means of heart rate assessments. The CBT + PA group showed a significantly stronger reduction in SAD symptomatology in comparison with the CBT plus education group.

In the study by Jelalian et al. (2011), the intervention was also CBT combined with additional exercise (CBT + PA). The intervention consisted of 16 1-h weekly treatment sessions during which adolescents and parents attended separate concurrent meetings and four biweekly maintenance sessions. In the control group, participants received peer-enhanced adventure therapy. Adolescents in both conditions were prescribed a balanced deficit diet with gradually increasing PA. Both groups demonstrated significant reductions in the perception of SA symptoms over time, but no significant group differences were found.

#### Meta-Analysis

##### Participants (Meta-Analysis)

The total number of subjects in the *n* = 4 studies, which were included in the meta-analysis, was 749 with a median of 52 per study and a range of 30–449. The studies were conducted between 2010 and 2020. In one of the studies, a clinical population with SAD diagnosis was described, and in *n* = 3 studies, a non-clinical population was described. The mean age of the study participants was 14 years (*SD* = 9.2). Age ranged from 9 to 32 years. Data from children in three studies (Hartmann et al., 2010; Lokös et al., 2013; Yu et al., 2020) and from young adults in one study (Jazaieri et al., 2012) were reported; 46.3% of the population in the studies were female (range from 20.5 to 54%).

##### Risk-of-Bias Assessment

**Figure 2:**
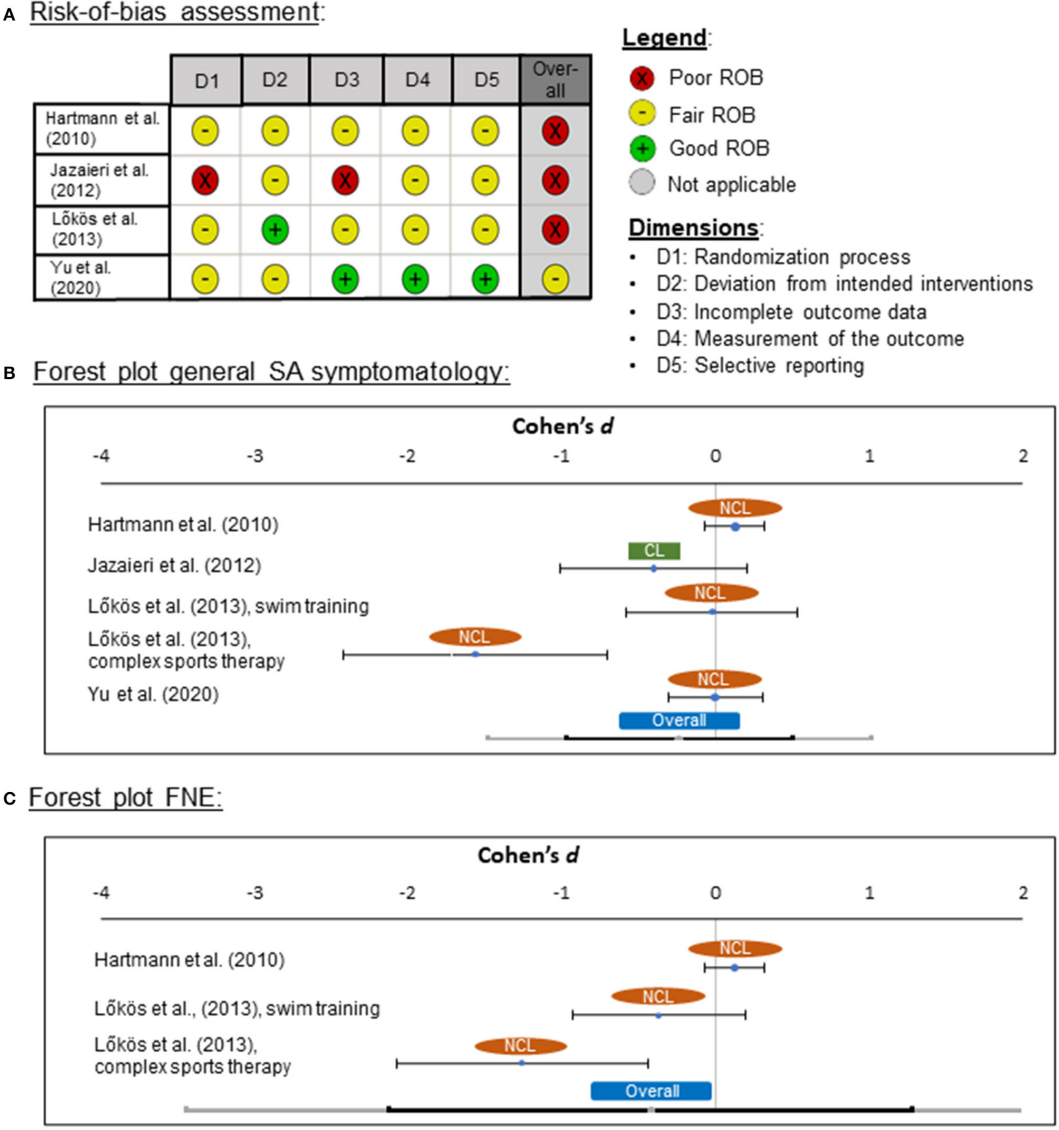
**(A)** Risk-of-bias (ROB) quality ratings for the randomized-controlled trials (RCT), **(B)** forest plot for general social anxiety (SA) symptomatology for clinical (CL) and non-clinical (NCL) samples, and **(C)** for fear of negative evaluation (FNE). ROB was assessed by means of the “Quality Assessment Tool of Controlled Intervention Studies” from the National Heart, Lung, and Blood Institute (2019). Negative effect sizes indicate that symptomatology was lower in the PA treatment than in the control group.

Risk of bias was rated for all four studies which were included in the meta-analysis. An overview about these study quality ratings is provided in Figure 2A. From these four RCTs, three were categorized as poor ROB and one as fair ROB. No study was found that was rated as high quality (i.e., good ROB). One reason for these ratings was that a power analysis was reported in one study only (Yu et al., 2020).

##### Effects of Physical Activity Interventions on Social Anxiety Symptoms

For the meta-analysis, Cohen's *d* was used as a measure for effect sizes, in which the mean of SA symptomatology after the treatment was compared between the intervention and the control groups. As a control group in the study of Jazaieri et al. (2012), the untreated SAD group was used instead of the MBSR group. Effect sizes ranged between *d* = −1.56 and *d* = 0.13 in the four RCTs (one clinical and three non-clinical trials). The pooled effect was medium in size (*d* = −0.24) and not significantly different from zero [*z*_(749)_ = −0.88, *p* = 0.377; Figure 2B]. Although the result was not significant, the pooled effect pointed in the expected direction that SA symptoms were lower in the PA group than in the control group. The pooled effect showed heterogeneity of variance (*Q* = 17.45, *p* = 0.002), suggesting the presence of one or more moderators.

In an additional meta-analysis, we examined the effects of exercise interventions on FNE symptoms. Two studies were included (Hartmann et al., 2010; Lokös et al., 2013) with effect sizes ranging from *d* = −0.90 to *d* = 0.13. The pooled effect was medium in size (*d* = −0.48) and not significantly different from zero [*z*_(531)_ = −1.42, *p* = 0.155; Figure 2C]. Again, the pooled effect showed heterogeneity of variance (*Q* = 13.34, *p* = 0.001).

##### Associations With Moderator Variables and Subgroup Analyses

Additional analyses showed no significant associations with the moderator variables age (*p* = 0.696) and sex (*p* = 0.488). It was not possible to conduct a moderator analysis for BMI, because BMI data were reported in two studies only. Because we found one clinical study only (Jazaieri et al., 2012), we could not conduct a subgroup analysis to compare clinical and non-clinical populations. Because overall ROB was comparable (high) in all four studies, no separate analysis for good or fair rated studies was conducted. Furthermore, no data on stress were reported, and therefore, the association between stress and treatment efficacy could not be investigated. Because of the high heterogeneity in reported interventions, subgroup analyses for different types of PA were also not possible.

### Interim Discussion Part 1 (Randomized Controlled Trials)

Overall, one RCT was found only in which all inclusion criteria were met, and a clinical sample was investigated (Jazaieri et al., 2012). Three additional studies were found, in which non-clinical samples were enrolled in RCTs. Combining these studies yielded no significant difference in SA symptomatology or FNE between the PA treatment and control group after the intervention. However, negative effects of medium effect size were found for both, general SA symptomatology and FNE, confirming that the effect pointed in the direction of lower SA or FNE in the PA treatment groups, suggesting potential benefits of PA interventions for the treatment of SAD or subclinical levels of SA. Generally, due to the low number of studies, a lack of power of the meta-analysis is present and a significant effect might not be detected. This is clearly pointing to missing high-quality studies in this research field.

Another reason for the non-significant effect of PA interventions on SA in our meta-analysis may—besides the lack of statistical power—be based on the inclusion of non-clinical samples. Furthermore, even in the clinical sample, low levels of SAD symptomatology and FNE were reported. One reason why participants with severe SAD or high levels of FNE were not included might be that for participating in a longtime PA intervention, a minimum level of motivation, open-mindedness, and communication skills is required, which can reinforce avoidance of such interventions for people with severe SAD.

One further limitation of our meta-analysis is that the only clinical RCT did not provide insight in the randomization process and it is, therefore, not clear whether it can be considered as a “real” RCT, or rather as a study with a non-randomized treatment–control group design. Furthermore, we had to change the initial intervention and control group assignments to fit our research question. Further limitations with respect to all four studies are that the interventions and control groups were very heterogeneous. Interventions consisted of different types of PA and durations, ranging from 7 days to 18 months. Furthermore, the control interventions were not comparable between the studies (CBT, MBSR, no treatment, usual practice with no intervention). Only one RCT (Yu et al., 2020) reported a power analysis, and therefore, we cannot decide if the sample sizes were overall large enough to be able to detect a difference in the main outcome of the effect of PA on SAD between the different groups.

Because of the low number of studies and, therefore, too low statistical power, further research is needed, in which the associations between SAD symptomatology and PA treatments are investigated and compared to non-PA treatment efficacy in studies with RCT designs in clinical settings, including people with severe SAD symptomatology.

## Part 2: Longitudinal Studies

### Introduction

Because of the low number of studies that were found in part 1, we decided to conduct a further meta-analysis, in which we also included longitudinal designs without a control group. The aims were to investigate whether PA interventions are suitable for changing SAD symptomatology in general and whether longitudinal changes in everyday PA levels are associated with changes in SA.

### Materials and Methods

#### Protocol and Registration

This meta-analysis has not been preregistered and was initiated after the analysis of part 1 was completed.

#### Search Strategy and Databases

The search strategy was the same as for the RCTs and the search results from part 1 were screened again.

#### Article Selection and Data Extraction

Article selection and data extraction were similar to the procedure for the RCTs with the difference that less strict criteria for the study design were used and all studies with a longitudinal design were considered, including the RCTs that were reported above.

#### Risk-of-Bias Assessment

For ROB assessment, the “Quality Assessment Tool for Before-After (Pre-Post) Studies With No Control Group” from the National Heart, Lung, and Blood Institute (2019) was used. Studies with an RCT design that were included in both analyses were rated again.

#### Statistical Data Analysis

As a measure for effect size for the meta-analysis, Cohen's *d* was considered. If not reported in the study, Cohen's *d* was calculated according to the following formula:

$$\begin{array}{l} \;d\left(longitudinal\right)=\;\frac{M\left(post\right)-M\left(pre\right)}{\sigma }\\ \text{with }\sigma =\sqrt{\frac{\left[n\left(post\right)-1\right]\times {SD\left(post\right)}^{2}+\left[n\left(pre\right)-1\right]\times {SD\left(pre\right)}^{2}}{\left[n\left(post\right)+n\left(pre\right)-2\;\right]}}. \end{array}$$

If necessary, *d* was derived from *r* as

$$\begin{array}{l} d=\frac{2r}{\sqrt{1-r^{2}}} \end{array}$$

(Fritz et al., 2012). The outcome measure was general SA symptomatology. No additional analyses for FNE or further core symptoms of SA were conducted. The other steps of the statistical analysis were almost the same as for part 1.

### Results

#### Overview of Search Results

With the new, less strict eligibility criteria, *n* = 28 studies were found, which met the inclusion criteria, from which *n* = 12 were included in the meta-analysis (Figure 1B). Two of these were RCTs, which were also included in part 1 (Jazaieri et al., 2012; Yu et al., 2020). The other two RCTs (Hartmann et al., 2010; Lokös et al., 2013) could not be included because the relevant data were not reported. From the further studies, in *n* = 5 studies, a PA intervention was reported (Lamarche et al., 2009; Lamarche and Gammage, 2010; Mamen et al., 2011; Shin and Lee, 2018; Luna et al., 2019). In one of these studies (Luna et al., 2019), additional data from a control group were available. However, because the design was not an RCT, this study was excluded in part 1 and the control group was not considered further. The other *n* = 5 studies reported longitudinal data with at least two measurement time points at which both PA and SA were assessed (Dimech and Seiler, 2011; ten Have et al., 2011; Jamal et al., 2012; Brière et al., 2018; Zink et al., 2019). An overview about the characteristics of the studies that were included in the meta-analysis is provided in Table 1 for the RCTs and in Table 2 for the further studies with longitudinal designs.

**Table 2:** Characteristics of the studies with a longitudinal design and which were included in the meta-analysis.

**References**	**Population**	**Main diagnosis**	***N***	***N* (post)**	**Dropout rate (%)**	**Mean age (SD)**	**Sex (% female)**	**Ethnicity**	**Education/** **employment**	**SA** **assessment**	**PA** **assessment**	**Intervention**	**Frequency PA intervention**	**Duration/ intensity single PA unit**	**Total duration PA intervention**
Brière et al. (2018)	NCL	NA	17,750	17,750	38	14.4 (1.3)	54	Canadian-born Caucasian (85%), Aboriginal (1%), Caribbean (2%), Latin American (2%), African (except Maghreb) (1%), Maghreban (1%), East-Asian (1%), South-East Asian (1%), South-Asian (2%), Middle-Eastern (1%), West-European (2%), East-European (1%)	Middle school to high school students	SCAS MASPAQ	“Do you regularly take part in an organized sport”; answer categories: “No, I don't participate in any sport,” “yes, once a week,” “yes, twice a week,” “yes, three times a week,” “yes, four or more times a week”	NA	NA	NA	NA
Dimech and Seiler (2011)	NCL	NA	208	145	15	7.72	51	Swiss nationals (87%)	Primary school students	SPAI-C	Extracurricular sport	NA	NA	NA	NA
ten Have et al. (2011)	NCL	NA	7,076	7,076	21	NR	49.4	Dutch	Primary education (6.3%), lower secondary education (36.5%), higher secondary education (29.3%), higher professional education (university degree, 27.9%)	CIDI	PA was assessed at baseline with the question: “How many hours per week have you engaged in physical exercise/ sport lately?”	NA	NA	NA	NA
Jamal et al. (2012)	CL	Current diagnosis of depression and/or an anxiety disorder	2,981	1,725	18	38.4 (13.00)	70.1	NR	Basic education (6.6%), intermediate education (61%), higher education (32.4%)	CIDI	IPAQ	NA	NA	NA	NA
Lamarche et al. (2009)^*^	NCL	NA	59	51	14	20.47 (1.99)	100	Caucasian	University students	SSAS SPAS-S	NA (intervention)	Step aerobic class	NR	7 min warm up + 20 min step aerobic + 7 min cool down	7 times
Lamarche and Gammage (2010)^*^	NCL	NA	87	84	NR	19.82	100	NR	University students	SSAS SPAS-S	NA (intervention)	Exercise	Once	30 min exercise	Once
Luna et al. (2019)^*^	NCL	NA	113	113 (*n*_int_ = 69, *n*_con_ = 44)	NR	13.82 (0.79)	43	NR	Secondary education students	SAS-A	NA (intervention)	Physical sports program	2–3 sessions per week	55 min	6 weeks
Mamen et al. (2011)^*^	CL	Main diagnosis of substance abuse/ dependence and comorbid psychiatric disorders according to DSM-IV	33	33	40	31.2 (9.9)	21.2	NR	NR	BSPS	Training was recorded in an individual training diary and heart rate was recorded for some exercises	11-month intervention	Daily	Overall: mean = 301 h (SD = 16 h) of training during the duration of the project; NR for individual sessions	11 months
Shin and Lee (2018)^*^	CL	SAD	60	48	20	35.45 (3.83)	0	Korean	Office workers	SPS	NA (intervention)	Circuit training program	3 times/week	70 min	8 weeks
Zink et al. (2019)	NCL	NA	2,521	2,521	20	14.6 (0.40)	56	47% Hispanic	High school students	RCADS	One item from the YRBSS, “During the past 7 days, how many days were you physically active for a total of at least 60 min per day? (Add up all the time you spent in any kind of physical activity that increased your heart rate and made you breathe hard some of the time)”	NA	NA	NA	NA

*Besides these studies, two randomized controlled trials from part 1 were included (Jazaieri et al., 2012; Yu et al., 2020), for which the study characteristics are reported in Table 1*.

*BSPS, The Brief Social Phobia Scale (Davidson et al., 1997); CIDI, The Composite International Diagnostic Interview (WHO, 1990); CL, clinical; DSM-IV, Diagnostic and Statistical Manual of Mental Disorders, fourth edition (American Psychiatric Association, 1994); IPAQ, The International Physical Activity Questionnaire (Craig et al., 2003); MASPAQ, The Questionnaire on the Social and Personal Adjustment of Quebec Adolescents (Le Blanc et al., 1996); N, sample size; NA, not applicable; NCL, non-clinical; n_con_, sample size in the control group; n_int_, sample size in the intervention group; NR, not reported; PA, physical activity; RCADS, The Revised Children's Anxiety and Depression Scale (Chorpita et al., 2000); SA, social anxiety; SAD, social anxiety disorder; SAS-A, the Social Anxiety Scale for Adolescents (La Greca and Lopez, 1998); SCAS, Spence Children's Anxiety Scale (Spence, 1998); SD, standard deviation; SPAI-C, The German version of the Social Phobia and Anxiety Inventory for Children (Beidel et al., 1995; Beidel, 1996); SPAS-S, The State Social Physique Anxiety Scale (Kruisselbrink et al., 2004); SSAS, The State Social Anxiety in Exercise Classes Scale (Martin and Fox, 2001); SPS, The Social Phobia Scale (Mattick and Clarke, 1998); YRBSS, the Youth Risk Behavior Surveillance System (Eaton et al., 2010)*.

* *Studies that included an intervention*.

#### Participants

The total number of subjects in the *n* = 12 studies, which were included in the meta-analysis, was 29,333 with a median of 84 per study and a range of 11 to 17,550. The studies were conducted between 2009 and 2020. The population of these studies was clinical in four studies and non-clinical in eight studies. The mean age of the study participants was 21 years (*SD* = 10.7). Age ranged from 8 to 38 years. In the study by ten Have et al. (2011), age groups were reported only. Therefore, this study was not included in the moderator analyses, in which age effects were investigated; 51.4% of the population in the studies were female (range from 0 to 100%). Ethnicity of the participants was mostly Caucasian, followed by Asian and Hispanic. The amount of regular PA before the intervention was reported in two studies only. In Lamarche et al. (2009), participants exercised 3.6 times per week (*SD* = 1.8) on average and reported that they had participated in 2.1 (*SD* = 1.6) step aerobic classes in their lifetime. In Lamarche and Gammage (2010), only women, who exercised two or fewer times per week in the past 6 months, were recruited. Of those who reported being physically active, mean exercise frequency was 1.1 times per week (*SD* = 0.8).

#### Types of Intervention and Physical Activity Assessment

In the study by Lamarche et al. (2009), the intervention consisted of a one-time step aerobic class. SAD symptomatology decreased after this exercise intervention. In Lamarche and Gammage (2010), the intervention was a 30-min exercise ball class, which was designed for beginners. SAD symptomatology was lower after the exercise intervention than before.

In the study by Luna et al. (2019), a physical sports program based on the sport education model by Siedentop et al. (2019) over 16 sessions was conducted. The program took place during the second school term. Guidelines from Hastie and Casey (2014) were followed for the design and validation of the program. Although SAD symptoms were lower after the intervention, this effect was not statistically significant.

In the study by Mamen et al. (2011), an 11-month training was conducted with a training partner on an almost daily basis. Intensity zones were based on the results from a baseline lactate profile test, and exercises were designed based on these findings and heart rate measurements to avoid too low-intensity training sessions. The training focused on improving aerobic capacity/power and endurance strength. Jogging, cycling, cross-country skiing, and swimming were some of the individual sports activities offered. In addition, group activities, such as aerobics, mountain hiking, and ball games, were also provided. The participants took part in several competitions for recreational athletes, both individually and in teams. The PA intervention led to a decrease in SAD symptoms.

The intervention in the study of Shin and Lee (2018) consisted of a circuit training program, which was constructed based on the exercise program applied in a previous research by this group (Nahm et al., 2015). The circuit training program significantly improved SAD symptomatology.

In the studies by Dimech and Seiler (2011), ten Have et al. (2011), Jamal et al. (2012), Brière et al. (2018), and Zink et al. (2019), no specific interventions were conducted, but longitudinal data on PA changes were reported. In all of these studies, data were assessed by means of self-report questionnaires (Table 2). ten Have et al. (2011), Jamal et al. (2012), Brière et al. (2018), and Zink et al. (2019) found that increases in PA over time were associated with reduction in SA. In the study by Dimech and Seiler (2011), a reducing effect of PA on SA was found for team sports only, but not for individual sports.

#### Risk-of-Bias Assessment

**Figure 3:**
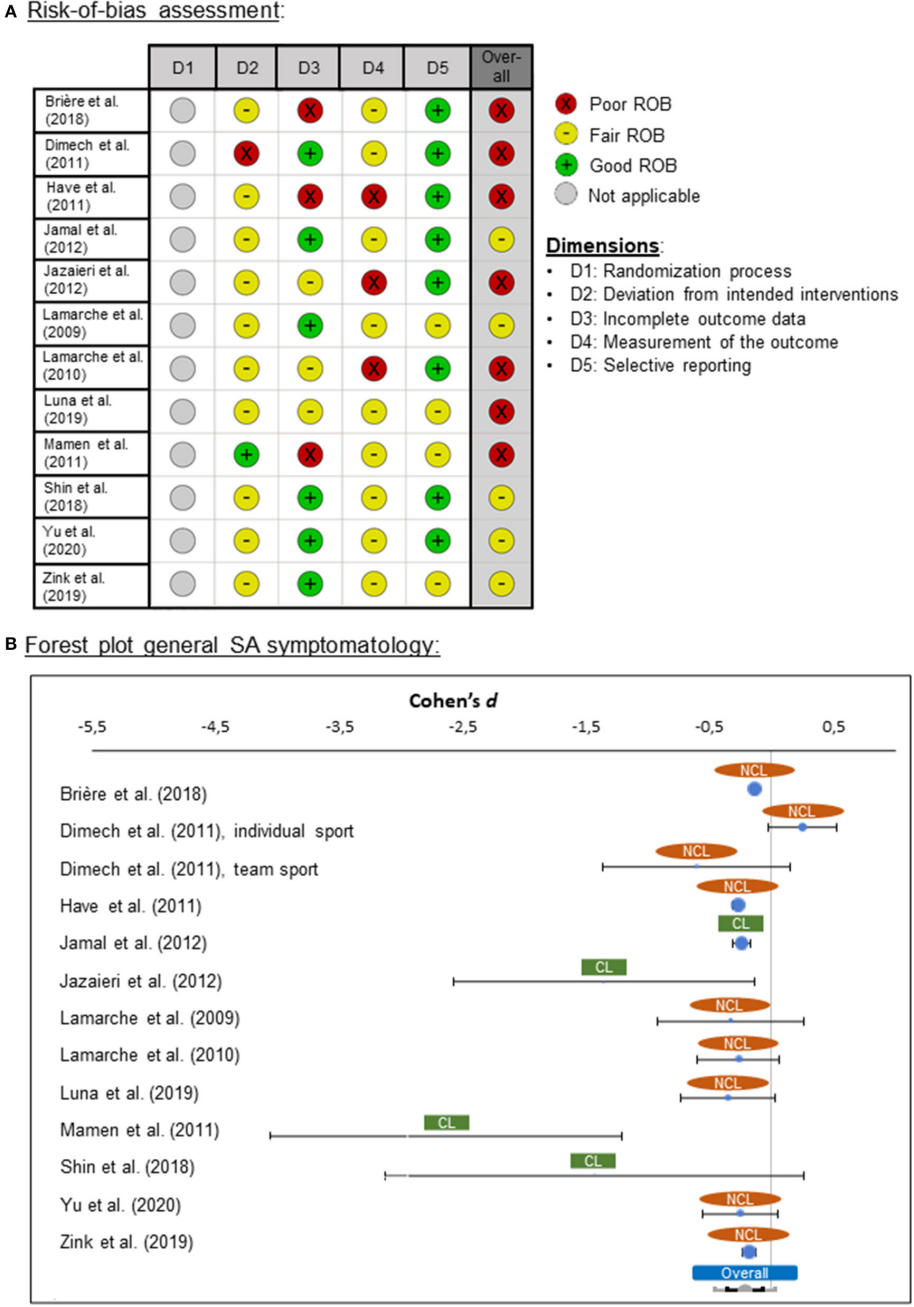
**(A)** Risk-of-bias (ROB) quality ratings for the longitudinal studies, **(B)** forest plot for general social anxiety (SA) symptomatology for clinical (CL) and non-clinical (NCL) samples. ROB was assessed by means of the “Quality Assessment Tool for Before-After (Pre-Post) Studies With No Control Group” from the National Heart, Lung, and Blood Institute (2019). Negative effect sizes indicate that symptomatology was lower at the second measurement time point (post-intervention) than at the first time point (pre-intervention).

From the longitudinal studies, seven were categorized as poor ROB and five as fair ROB. No study was found that was rated as high quality (i.e., good ROB; Figure 3A). One reason for these ratings was that in none of the studies was a power analysis reported.

#### Meta-Analysis

##### Effects of Exercise Intervention on Social Anxiety Symptoms

The general effects of PA interventions on SA in the 12 studies showed effect sizes ranging from *d* = −2.64 and *d* = 0.25. The pooled effect was medium in size (*d* = −0.22) and significantly different from zero [*z*_(29,333)_ = −3.53, *p* = 0.001], pointing in the direction of lower SA after PA treatments (Figure 3B). The pooled effect showed heterogeneity of variance (*Q* = 74.67, *p* < 0.001), suggesting the presence of one or more moderators.

##### Associations With Study Quality

To test whether this finding is due to ROB, which was rated as high in 7 of the 12 studies, we repeated the analysis for the fair rated studies. The results did not show a meaningful difference in comparison with the meta-analysis, where all studies were included. The effect sizes for the five fair rated studies ranged from *d* = −1.43 to *d* = −0.18 and, again, in the direction of lower SA after PA treatments. The pooled effect was again medium in size (*d* = −0.21) and significantly different from zero [*z*_(4,385)_ = −7.68, *p* < 0.001].

##### Associations With Moderator Variables and Subgroup Analyses

An additional analysis showed a significant association with the moderator variable age (*p* = 0.003; range: 7–39 years). Adults showed a larger effect of PA on SA than children and adolescents. Sex and BMI were no significant moderators (*p* = 0.246 and *p* = 0.603). Furthermore, we conducted a subgroup analysis for clinical vs. non-clinical populations. The results indicated that both groups were very heterogeneous (clinical: *Q* = 17.84, *p* < 0.001; non-clinical: *Q* = 52.61, *p* < 0.001) and, hence, cannot be meta-analyzed as if it were one single population. There were only three studies describing comorbidities. The percentage of patients with comorbidities as a moderator was significant (*p* < 0.001), indicating that those who suffered from a comorbid disorder showed stronger effects of PA on SAD symptoms.

We could not conduct a meta-analysis for stress as a moderator between PA and SA symptoms, as stress was reported in two studies only, which present different study designs and used different stress measures (Mamen et al., 2011; Jazaieri et al., 2012). Because of the high heterogeneity in the reported interventions (*n* = 5), subgroup analyses for different types of PA were also not possible.

Furthermore, we conducted subgroup analyses to compare those studies that included an intervention and those that assessed PA by self-reported methods (e.g., questionnaires). In the intervention subgroup, the general effects of exercise on SA symptoms in the seven studies showed effect sizes ranging from *d* = −2.64 to *d* = −0.25. The pooled effect was strong (*d* = −0.55) and significantly different from zero [*z*_(343)_ = −2.22, *p* = 0.027]. However, this effect did not survive our adjustment for multiple comparisons (α_adjusted_ = 0.0071). The pooled effect showed heterogeneity of variance (*Q* = 16.40, *p* = 0.012), suggesting the presence of one or more moderators. In the non-intervention subgroup, in which daily PA behavior was assessed, the effects of regular PA on SA in the seven studies showed effect sizes ranging from *d* = −0.61 to *d* = 0.25. The pooled effect was small (*d* = −0.18) and significantly different from zero [*z*_(28,990)_ = −3.06, *p* = 0.002]. The pooled effect showed heterogeneity of variance (*Q* = 53.83, *p* < 0.001), suggesting the presence of one or more moderators. Therefore, the effect of PA on SA could be confirmed for the non-interventional studies, and a marginally significant effect in the same direction was found for the interventional studies. However, the strength of the effect was stronger for the interventional than for the non-interventional studies.

### Interim Discussion Part 2 (Longitudinal Studies)

Overall, 12 studies with longitudinal designs were found, from which data from a clinical sample in four studies and from a non-clinical sample in eight studies were reported. Our meta-analysis yielded a highly significant effect of medium size for the association between changes in SA symptomatology after PA treatment or PA in everyday life in comparison with the first measurement time point. Therefore, we could confirm the beneficial effects of regular PA on SA.

Our results indicate that there are moderators responsible for the significant outcome between PA and SA. Therefore, we examined the result for potential moderators, and the moderators age and comorbidity became significant. Furthermore, although we could not analyze this statistically because of too high heterogeneity, our findings point in the direction that the positive effects of PA on SA are stronger in clinical populations.

Most of the studies were of a prospective design and sample sizes ranged from small (*n* = 33) to very large (*n* = 17,750). Power analyses were not reported in most studies, and many of the studies were rated as poor quality (i.e., high ROB). Furthermore, there was a high heterogeneity across all longitudinal studies. Different SA and PA assessments were used, and assessments were mostly based on self-reports. This could have led to further bias especially for the assessment of PA, because the instruments ranged between one-item and standardized questionnaires. Furthermore, participants with severe SAD and higher levels of SA and those who were not engaging in PA before the intervention were more likely to be lost to follow-up, leading to an additional bias. Therefore, generalizability of our findings is questionable, and more research is needed.

## Part 3: Cross-Sectional Studies

### Introduction

To get a comprehensive overview, we decided to conduct a further search, in which we looked for cross-sectional studies in which PA and SA (clinically relevant or subclinical) were assessed. The aim was to investigate whether a general association between SA and the amount of PA can be found. Furthermore, we aimed to investigate whether this association differs between clinical and non-clinical samples and whether it is related with the above mentioned potential moderator variables.

### Materials and Methods

#### Protocol and Registration

This meta-analysis has not been preregistered and was initiated after the analysis of part 2 was completed.

#### Search Strategy and Databases

The search strategy was nearly the same as for parts 1 and 2 with the difference that block 3 (referring to the intervention terms) was excluded. The search was conducted in August 2020.

#### Article Selection and Data Extraction

Article selection and data extraction were similar to the procedures for parts 1 and 2 with the difference that the study design was cross-sectional.

#### Risk-of-Bias Assessment

For ROB assessment, the “Quality Assessment Tool for Observational Cohort and Cross-Sectional Studies” from the National Heart, Lung, and Blood Institute (2019) was used.

#### Statistical Data Analysis

As a measure for effect sizes for the meta-analysis, Pearson correlation coefficients *r* were used. We considered correlation coefficients *r* between 0.1 and 0.3 as small effects, between 0.3 and 0.5 as medium effects, and >0.5 as strong effects (Cohen, 1988). If necessary, *r* was derived from *d* as

$$\begin{array}{l} r=\frac{d}{\sqrt{d^{2}+4}} \end{array}$$

(Fritz et al., 2012). When odds ratios (OR) were reported, these were transformed to *d* according to

$$\begin{array}{l} d=\ln\left(OR\right)\times \frac{\sqrt{3}}{\pi } \end{array}$$

(Borenstein et al., 2011) and afterwards to *r*. The other steps of the statistical analysis were the same as for parts 1 and 2. Additionally, a subgroup analysis in which team sports were compared to individual sports was conducted. For this comparison, Cohen's *d* between mean SA between participants who engaged in individual and who engaged in team sports was calculated according to

$$\begin{array}{l} \;d\left(individual\;vs.\;team\right)=\;\frac{M\left(individual\right)-M\left(team\right)}{\sigma }\\ \text{with }\sigma =\sqrt{\frac{\left[n\left(individual\right)-1\right]\times {SD\left(individual\right)}^{2}+\left[n\left(team\right)-1\right]\times {SD\left(team\right)}^{2}}{\left[n\left(individual\right)+n\left(team\right)-2\;\right]}}, \end{array}$$

with positive values indicating higher SA for individual than for team sports and negative values reflecting lower SA for individual than for team sports.

### Results

#### Overview of Search Results

**Figure 4:**
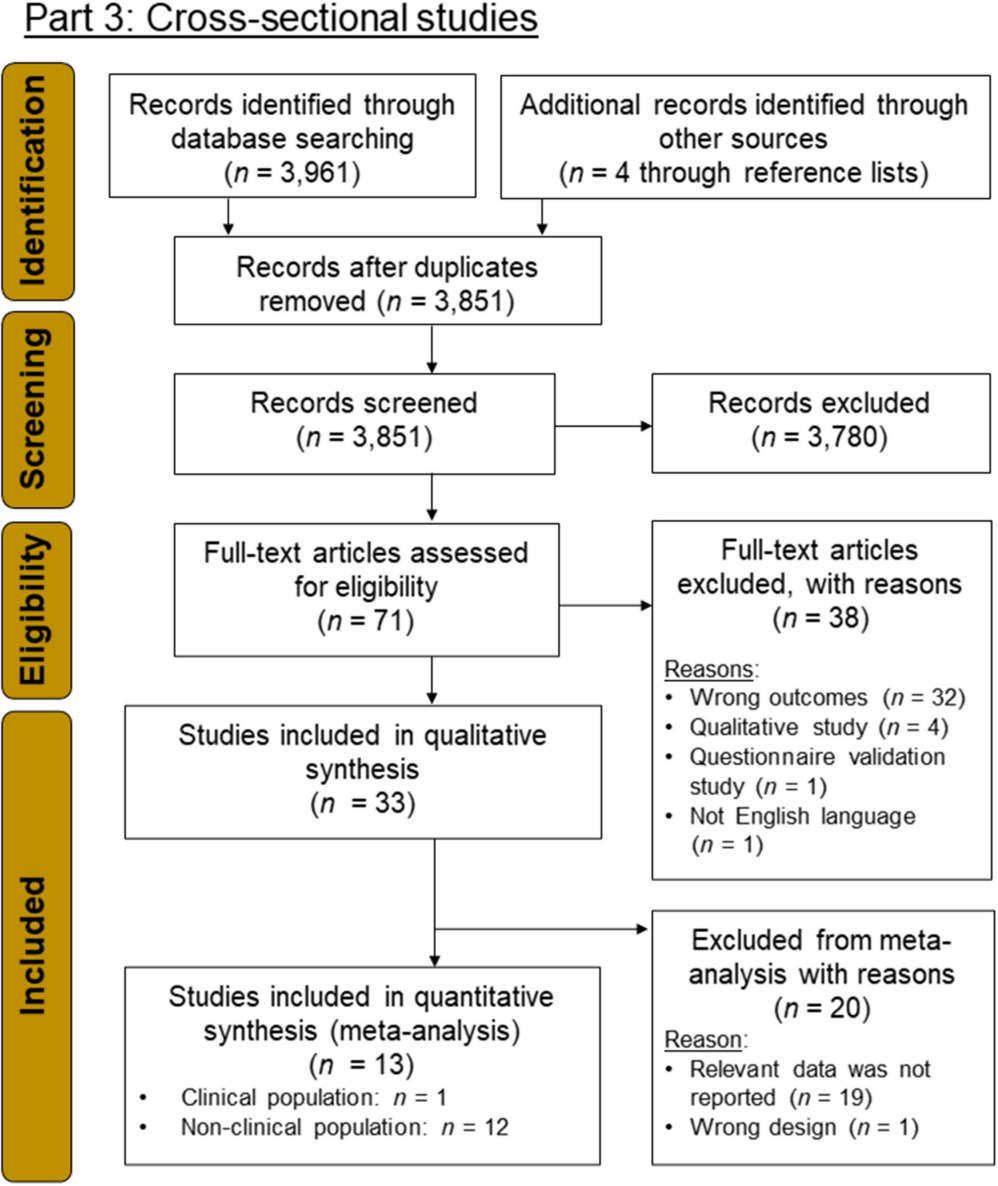
PRISMA flow-chart diagram (Moher et al., 2009) for part 3 in which cross-sectional studies were searched for.

With this new search strategy, *n* = 33 studies were found, which met the eligibility criteria, from which *n* = 13 studies were included in the meta-analysis (Figure 4). An overview about study characteristics is provided in Table 3.

**Table 3:** Characteristics of the cross-sectional studies that were included in the meta-analysis.

**References**	**Population**	**Main diagnosis**	***N***	**Mean age (SD)**	**Sex (% female)**	**Ethnicity**	**Education**	**SA assessment**	**PA assessment**	**Control group treatment**
Ashdown-Franks et al. (2017)	NCL	NA	781	20.34 (0.71)	55.2	NR	High school students	Two items: (i) “A time when you felt very afraid or really shy meeting new people, going to parties, going on a date” and (ii) “A time when you felt very afraid or uncomfortable when you had to do something in front of a group of people (giving a speech, speaking in class)”	IPAQ	NA
Atalay and Gençöz (2008)	NCL	NA	118	20.07 (1.63)	100	NR	Higher education degree or college diploma	LSAS-SR	Time and mode of physical exercise	NA
Benau et al. (2020)	NCL	NA	297	19.3 (2.24)	63.4	Caucasian (79%), African-American (12%), Hispanic (3%), Asian/Pacific Islander (2%), Native American/Native Alaskan (<1%), other (2%)	Higher education degree or college diploma	SPIN	Athletic participation	NA
Dimech and Seiler (2010)	NCL	NA	201	7.72 (0.67)	50.8	Swiss nationals (88.6%) or possessed dual citizenships	Primary school students	SPAIK	Sport participation	NA
Dong et al. (2018)	NCL	NA	4,334	<18 or ≥18 years old	53.6	NR	High school students	LSAS-SR	Physical exercise (days/week) was divided into three grades: 1) 0 day, 2) 1~3 days, and 3) >3 days.	NA
Goodwin (2003)	NCL	NA	5,876	15–54	49.8	Caucasian (75.1%), African-American (11.9%), Hispanic (9.5%), other (3.5%)	Less than 8th grade education (19.3%), 9th−11th grade education (35.2%), high school diploma (22%), college degree (23.6%)	CIDI	Verbal queries “How often do you get physical exercise—either on your job or in a recreational activity?” Response choices were: “regularly,” “occasionally,” “rarely,” and “never.”	NA
Herring et al. (2014)	CL	SA: *n* = 375; 16% reported the use of psychotropic medication.	1,036	19.67 (2.95)	100	Caucasian (78%), Asian (9%), African-American (8%), Hispanic/Latino (3%), multiracial (2%)	Higher education degree or college diploma	PDSQ	7dPAR	NA
Kim et al. (2020)	NCL	NA	2230	14.58 (NR)	49.3	NR	High school students	MINI-KID	Categories of PA: 6–7 days (0, reference), 4–5 days (1), 0–3 days (2)	NA
Koç and Dündar (2018)	NCL	NA	382	NR	49.7	NR	Secondary school students	SAS	PA was divided in doing and not doing sports	NA
Özşahin and Altinas (2018)	NCL	NA	224	18–56	100	NR	NR	LSAS-SR	Exercise for weight loss	NA
Ren and Li (2020)	NCL	NA	1,606	11.67 (1.94)	49.7	Asian	Primary school students	SASC	PARS-3	NA
Rocha et al. (2014)	NCL	NA	87	25.95 (1.75)	37.9	NR	Higher education degree or college diploma	K-MPAI	A questionnaire to assess PA habits	NA
Üstün and Yapici (2019)	NCL	NA	200	16.29 (1.11)	50	NR	High school students	SAS-A	The weekly duration/day of doing sports, the aim for doing sports	NA

*7dPAR, 7-Day Physical Activity Recall (Dishman and Steinhardt, 1988); CIDI, the Composite International Diagnostic Interview (WHO, 1990); CL, clinical (Connor et al., 2000); IPAQ, The International Physical Activity Questionnaire (Craig et al., 2003); K-MPAI, Kenny MPA Inventory Questionnaire (Rocha et al., 2011); LSAS-SR, Liebowitz Social Anxiety Scale-Self-Report (Liebowitz et al., 1985; Fresco et al., 2001); MINI-KID, Mini International Neuropsychiatric Interview for Children and Adolescents (Sheehan et al., 2010); N, sample size; NA, not applicable; NCL, non-clinical; NR, not reported; PA, physical activity; PARS-3, Physical Activity Rating Scale (Liang, 1994); PDSQ, the Psychiatric Diagnostic Screening Questionnaire (Zimmerman et al., 1999); SA, social anxiety; SAS, The Social Anxiety Scale (Özbay and Palanci, 2001); SASC, Social Anxiety Scale for Children (La Greca et al., 1988), originally adapted from the Social Anxiety Scale for Children–Revised (La Greca and Stone, 1993); SD, standard deviation; SPAIK, The German Version (SPAIK) of the Social Phobia and Anxiety Inventory for Children (SPAI-C; Melfsen et al., 2001); SPIN, Social Phobia Inventory (Connor et al., 2000)*.

#### Participants

The total number of subjects in the *n* = 13 studies was 16,801 with a median of 336 per study and a range of 87–5,876. The studies were conducted between 2003 and 2020. The population of the studies was clinical in one study and non-clinical in 12 studies. The mean age of the study participants was 18 years (*SD* = 4.9). Age ranged from 8 to 26 years; 63.6% of the population were female (range from 37.9 to 100%). Ethnicity of the participants was mostly Caucasian, followed by Asian and Hispanic.

#### Assessment of Physical Activity

Physical activity was assessed through standardized questionnaires (Herring et al., 2014; Ashdown-Franks et al., 2017; Ren and Li, 2020), such as the International Physical Activity Questionnaire (IPAQ; Craig et al., 2003), the 7-Day Physical Activity Recall (7dPAR; Dishman and Steinhardt, 1988), and the Physical Activity Rating Scale (PARS-3; Liang, 1994). In five studies (Atalay and Gençöz, 2008; Dimech and Seiler, 2010; Rocha et al., 2014; Üstün and Yapici, 2019; Benau et al., 2020), PA was assessed through self-constructed questionnaires or questions, such as “How often do you get physical exercise—either on your job or in recreational activity?” In four studies (Goodwin, 2003; Dong et al., 2018; Koç and Dündar, 2018; Kim et al., 2020), PA was divided into different categories, such as “doing” or “not doing sports” or time and mode of exercise.

#### Risk-of-Bias Assessment

**Figure 5:**
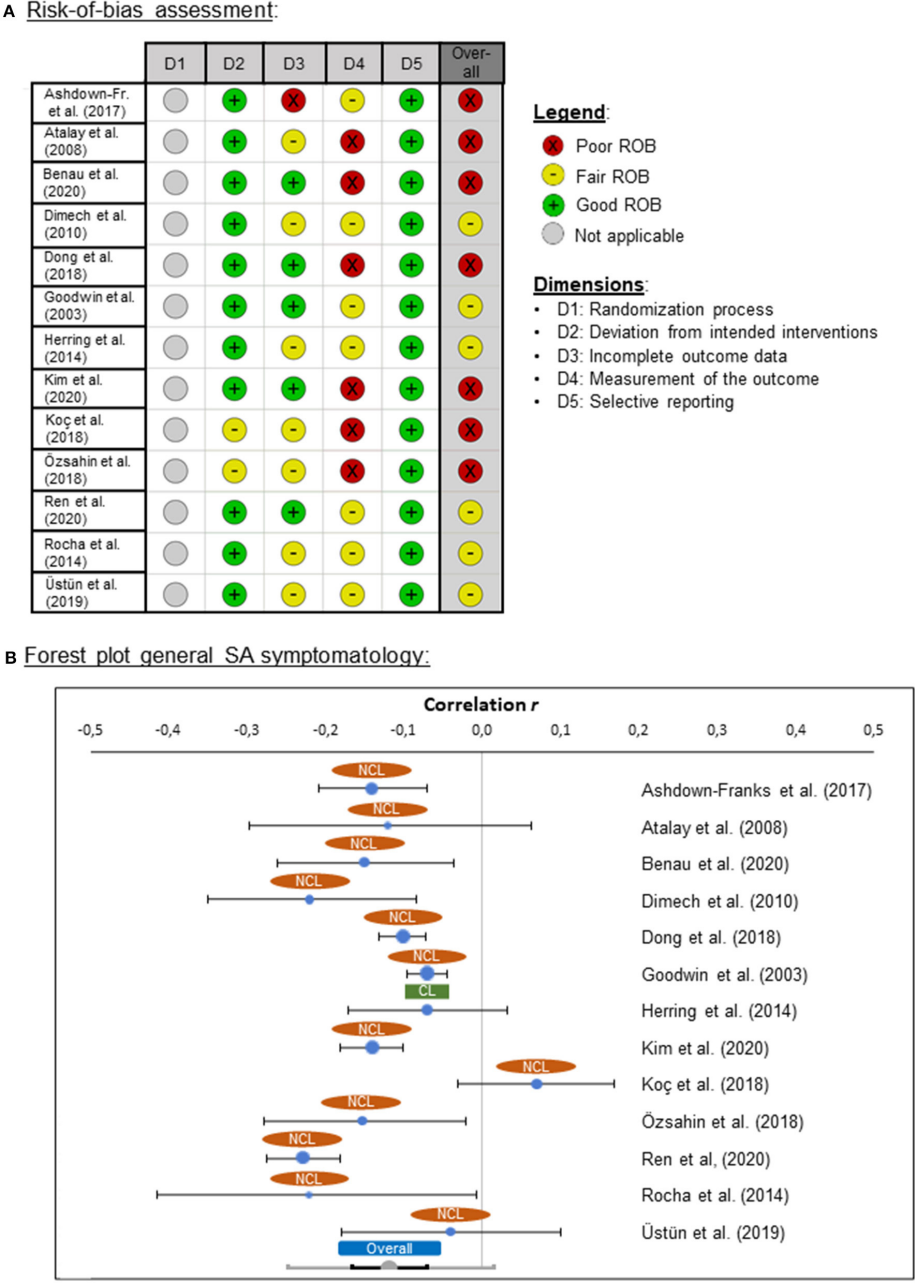
**(A)** Risk-of-bias (ROB) quality ratings for the cross-sectional studies, and **(B)** forest plot for general social anxiety (SA) symptomatology for clinical (CL) and non-clinical (NCL) samples. ROB was assessed by means of the “Quality Assessment Tool for Observational Cohort and Cross-Sectional Studies” from the National Heart, Lung, and Blood Institute (2019). Negative effect sizes indicate that higher levels of PA were associated with lower levels of SA.

From the 13 cross-sectional studies, seven were categorized as poor ROB and six as fair ROB. No study was found that was rated as high quality (i.e., good ROB; Figure 5A). Power analyses were reported in two studies only (Dimech and Seiler, 2010; Üstün and Yapici, 2019).

#### Meta-Analysis

##### Associations Between Social Anxiety and Physical Activity

The associations between SA and PA were examined in 13 studies with effect sizes ranging from *r* = −0.23 and *r* = 0.07. The pooled effect size was small (*r* = −0.12), but significantly different from zero [*z*_(16,801)_ = −5.34, *p* < 0.001], pointing in the direction that higher levels of PA were associated with lower levels of SA (Figure 5B). The pooled effect showed heterogeneity of variance (*Q* = 54.90, *p* < 0.001), suggesting the presence of one or more moderators.

##### Associations With Study Quality

To test whether this finding is due to ROB, which was rated as high in seven of the 13 studies, we repeated the analysis for the fair rated studies. The results did not show a meaningful difference in comparison with the meta-analysis that included all cross-sectional studies. The effect sizes ranged from *r* = −0.23 to *r* = −0.04 and the pooled effect size was small (*r* = −0.14), but significantly different from zero [*z*_(8,345)_ = −3.70, *p* < 0.001]. The pooled effect showed heterogeneity of variance (*Q* = 38.28, *p* < 0.001), suggesting the presence of one or more moderators.

##### Associations With Moderator Variables and Subgroup Analyses

Additional analyses showed no significant associations with the moderators age (*p* = 0.083) and sex (*p* = 0.841). Body mass index was reported in two studies only, and therefore, no moderator analysis for BMI could be conducted. Furthermore, a subgroup analysis for clinical vs. non-clinical populations was also not possible, because in one study a clinical population was reported only. The overall effect did not differ, when the clinical study was excluded from the meta-analysis [*r* = −0.12, *z*_(16,426)_ = −5.15, *p* < 0.001]. Furthermore, it was not possible to conduct an analysis with the moderator variable stress because no stress measure was reported in any of the studies.

Additionally, we conducted a meta-analysis on the effects of team sports vs. individual sports on SA. Four studies could be included into this analysis (Ashdown-Franks et al., 2017; Koç and Dündar, 2018; Üstün and Yapici, 2019; Benau et al., 2020). The effect sizes ranged from *d* = −0.09 to *d* = 0.15 with negative values indicating lower SA for individual than for team sports. The pooled effect size was small (*d* = −0.03) and not significantly different from zero [*z*_(1,330)_ = −0.65, *p* = 0.517], indicating that there was no difference in SA between individual and team sports. The pooled effect showed no heterogeneity of variance (*Q* = 4.01, *p* = 0.260), not indicating the presence of any moderators.

### Interim Discussion Part 3 (Cross-Sectional Studies)

Overall, a small negative association between SA and the amount of regular PA was found, indicating that higher levels of PA were associated with lower SA. Furthermore, the results indicate that there are moderators responsible for the significant outcome between PA and SA. Therefore, we examined the result for potential moderators, but none of the moderators we hypothesized (age and sex) became significant. For BMI and comorbidities, not enough data were reported. Therefore, further research on potential moderators is required. We did not find a difference in SA between individual and team sports, but four studies could be included in this analysis only, which all reported data for non-clinical samples.

Overall, the main limitation of the included studies is the cross-sectional design, and thus, causal inferences cannot be drawn. Therefore, whether the negative effect reflects that PA leads to a reduction of SA must be confirmed by means of longitudinal studies. Further limitations are that the sample sizes ranged from small to large, statistical power was not often reported, and the studies overall lacked in quality. Therefore, as for the RCTs and the longitudinal studies, the ROB rating resulted in only seven fair rated studies, and none was rated as good quality. There was a high heterogeneity across all the cross-sectional studies. Furthermore, several SA and PA assessment tools were used and were in most cases based on self-reports. This could have led to a bias especially in the assessment of PA, as the instruments used ranged from one question, divided into categories, to standardized questionnaires. There was only one clinical study, and therefore, it was not possible to compare clinical and non-clinical populations. Hence, generalizability of these findings is not possible.

## Overall Discussion

The aim of our systematic review and meta-analyses was to investigate whether a PA intervention is a suitable treatment for SAD. Furthermore, we aimed to investigate whether PA in general is associated with SA. Overall, we could confirm the association between PA and SA as well as the positive effects of PA treatments on SAD symptomatology and on subclinical levels of SA. However, high-quality studies with an RCT design were rare and one study was found only in which a PA intervention was conducted in a clinical SAD sample and compared with a control intervention.

For the studies with an RCT design (which included one clinical and three non-clinical samples), we did not find a significant difference in SA between the PA treatment and the control group after the interventions. However, this non-significance might be due to low statistical power because of the low number of included studies. Anyhow, the (non-significant) effect pointed in the expected direction and was of medium size (*d* = −0.24). Furthermore, we found the same direction of an (also non-significant) medium effect for FNE between the treatment and the control group (*d* = −0.48). For the longitudinal and cross-sectional studies, statistically significant associations between PA and SA were found, which were of medium and small effect sizes (longitudinal studies: *d* = −0.22; cross-sectional studies: *r* = −0.12). Higher levels of PA were associated with lower SA and PA treatments led to reduction in SA.

However, the pooled effect sizes showed heterogeneity of variance, suggesting the presence of one or more moderators. We tested the moderators age, sex, BMI, and comorbidities for all designs. Only for the longitudinal studies, the moderators age and percentage of patients with comorbidities were confirmed as significant moderator variables. Adults as well as people with comorbidities showed stronger benefits of PA on SA than adolescents or children as well as people without comorbidities. However, there was a bias toward clinical samples in adult populations and non-clinical people in children and adolescents in the studies that were included in our analysis, which might confound our result. Another reason for the smaller effect size in children might be that children are generally more active than adults and that baseline levels of PA were higher in this age group than in adults, which might have masked an effect of PA on SA. Whether the amount of regular PA before the treatment moderated efficacy could not be analyzed because baseline PA levels were reported in one RCT and in two longitudinal studies only. Moreover, because of the heterogeneity of interventions with regard to the number of sessions per week, overall duration, duration per session, and intensity per session, we were not able to create a convincing variable that could have been used in a moderator analysis to investigate whether the intensity or duration of the interventions was associated with efficacy.

Nevertheless, our findings show that PA interventions are especially suitable for adults with SAD diagnosis, which is particularly promising, because SAD often remains undiagnosed or untreated, because it is often attached to a personal trait rather than to a mental disorder. In many cases, SAD has an early onset during childhood or adolescence, and therefore, older patients often have a long history of SA. The most effective RCT was the complex sports therapy, although a non-clinical population was investigated. The reasons for this superior efficacy might be the high intensity of this therapy (2 weekly swim sessions of 60 min each, 1 weekly 60-min session of indoor sports, and one 60-min session of outdoor sports) as well as the diversity of the program which might have increased motivation.

Generally, it is especially important to identify and improve cost-effective interventions to prevent and treat SAD. Physical activity can easily be integrated in everyday life in every age group without major hurdles and is, therefore, promising for people with a lifetime history of SAD. Unfortunately, we could not find any prospective cohort studies which included a lifespan perspective from childhood, through adolescence to older adulthood. However, we found some studies, such as Brière et al. (2018), which have shown in a short time of 1 year that PA is a protective factor that contributes to psychological resilience in adolescents who are at increased risk for developing SAD. Therefore, we conclude that PA is a promising means of an intervention for every age group.

Initially, we planned to test stress as a moderator variable, because PA can lead to perceived and physiological stress reduction (Elosua et al., 2003; Rimmele et al., 2009; Wegner et al., 2014; Mücke et al., 2018) and stress reduction could have a positive effect on SAD (Herdt et al., 2013). Therefore, we expected to find more studies in this area to include in our meta-analysis. However, two studies in which perceived stress (but not physiological stress) was assessed were found only and it was not possible to analyze this association. Therefore, more research is needed, in which the associations between PA, SA, and physiological stress system activity are investigated. Furthermore, we aimed to compare the efficiency of different types of PA interventions. All studies that we found focused on aerobic or endurance exercises, and thus, our findings cannot be generalized to other forms of exercise. Therefore, future research is needed in which other types of exercise (e.g., resistance training) will be investigated. Resistance training seems to be particularly promising because it has been shown to be associated with stress reduction (Becker et al., 2020). Moreover, we could not conduct a moderator analysis for BMI for the RCTs and cross-sectional studies, because BMI data were reported in a too low number of studies. However, BMI seems to be particularly important because overweight can be associated with a higher risk for peer rejection or mobbing (Jelalian et al., 2011), which might counteract the positive effects of PA on SA. Therefore, BMI should be included in future research on the associations between PA and SA.

Although PA is often recommended in addition to CBT (Stathopoulou et al., 2006), we found two studies only in which CBT and a PA intervention were combined and compared with CBT plus another intervention (Merom et al., 2008; Jelalian et al., 2011). Nevertheless, the findings from these studies are promising because Merom et al. (2008) found a reducing effect of PA on SAD in addition to CBT in an RCT. Jelalian et al. (2011) also reported reductions in SA over time, but with no effect of treatment condition. Hence, there was no difference between the addition of PA to CBT and the peer-enhanced adventure therapy as an addition to CBT. Therefore, in future research, the potential of PA interventions as an additional treatment to CBT for SAD needs to be further investigated.

Further limitations are that there was a lack of uniformity in the measuring instruments, especially for PA assessment. For the RCTs, assessment tools varied from standardized questionnaires (Herring et al., 2014; Ashdown-Franks et al., 2017; Ren and Li, 2020) and self-constructed questionnaires to single items (Atalay and Gençöz, 2008; Dimech and Seiler, 2010; Rocha et al., 2014; Üstün and Yapici, 2019; Benau et al., 2020), or PA was divided into different categories, time, and modes of exercise (Goodwin, 2003; Dong et al., 2018; Koç and Dündar, 2018; Kim et al., 2020). For the longitudinal and cross-sectional studies, the instruments that were used for PA assessment also ranged from one-item assessments to standardized questionnaires.

Overall, most studies showed qualitative inadequacies for all three study designs, which resulted in fair and poor ROB ratings. Especially for the RCTs, we expected to find more high-quality trials. Unfortunately, we only found one RCT that we rated as fair ROB. Although the RCTs were in most cases rated as fair in the individual dimensions and, in some dimensions, two studies were even rated as good, this was not sufficient to consider them as good or at least as fair overall. One main reason for the poor ratings was that loss to follow-up was in most cases >20%. However, this might be due to the accessibility and reliability of the sample and was not necessarily due to the methodology of the studies. This might also be the reason why ROB was not better for the longitudinal and cross-sectional studies than for the RCTs. While there was no meaningful difference in the effect of PA on SAD, when fair rated studies were included only, we do not know if there would be a difference and possibly higher effect sizes if better rated studies were available.

Overall, we found only a few studies in which clinical samples were investigated. Even in the studies in which people with SAD diagnosis participated, the levels of SAD symptoms were not severe at baseline, and thus, participants with very high levels of SAD symptoms were missing in these studies. One reason for this might be associated with SAD symptomatology *per se*, because it requires motivation and a degree of open-mindedness to participate in an intervention and to attend to it regularly, which is a major challenge for people with SAD. A further hurdle that needs to be overcome is that often anxiety disorders are related with exercise anxiety (Mason et al., 2019), which might be another reason for dropout or a reason which prevents people with SA from engaging in regular PA. Nevertheless, we suggest including people with severe SAD symptomatology in future studies, although this might be challenging due to the nature of the disorder and to the expectation of high dropout rates. To potentially achieve this goal, studies should be conducted in clinical settings, rather than in everyday settings. Therefore, we recommend conducting future research on the associations between PA and SAD in clinics that are specialized on SAD.

## Conclusions

With this review and meta-analyses, we present a comprehensive systematic synthesis of the literature about the effects of PA on SA in a differentiated manner. We conclude that PA interventions are a promising means for an (additional) treatment of SAD or to reduce subclinical SA in non-clinical samples. However, much more research is needed in which high-quality studies with RCT designs are used and clinical samples are investigated. Furthermore, several questions with regard to potential moderating variables (e.g., age, sex, BMI, type of intervention, stress, comorbidities, and amount of regular PA before the intervention) remain open. Although research including this population is challenging because of high dropout rates, it is particularly important because SAD and subclinical SA are widespread and remain too often untreated.

## Data Availability Statement

The original contributions presented in the study are included in the article/Supplementary Material, further inquiries can be directed to the corresponding author/s.

## Author Contributions

MZ and LB designed the study, conducted the search, screened the search results, rated the risk of bias, conducted the meta-analyses, and wrote the manuscript.

## Conflict of Interest

The authors declare that the research was conducted in the absence of any commercial or financial relationships that could be construed as a potential conflict of interest.
