# Supplementary material for: Physical Activity as a Treatment for Social Anxiety in Clinical and Non-clinical Populations: A Systematic Review and Three Meta-Analyses for Different Study Designs
Source: Front Hum Neurosci. 2021 Jun 11;15:653108. doi: 10.3389/fnhum.2021.653108 (PMC8230570; doi:10.3389/fnhum.2021.653108)
Supplement: Supplementary file 1 [file Data_Sheet_1.docx]

**Supplementary Material S1: Search terms**

***Block 1***: ["Social phobi*" OR "social-phobi*" OR "social anxiety disorder" OR "social-anxiety disorder" OR "social anxiety-disorder" OR "social-anxiety-disorder" OR "social anxiety" OR "social neuros*" OR "social-neuros*" OR "social fear" OR "social-fear" OR "social anxious*" OR "generalized social phobia" OR "generalized-social phobia" OR "generalized social-phobia" OR "generalized-social-phobia" OR "specific social anxiety" OR "specific social phobia" OR sociophob* OR socio-phob* OR "socio phob*" OR anthropophob* OR "social awkwardness" OR "avoid* of social performance" OR "avoid* of social interaction" OR "avoid* of social activity" OR "avoid* of social affairs" OR "avoid* of public*" OR "performance anxiety" OR "performance-anxiety" OR shyness OR "escape and avoidance behavior*" OR "escape- and avoidance behavior*" OR "escape and avoidance-behavior*" OR "escape- and avoidance-behavior*" OR "social avoidance" OR "social isolation" OR "F40.1" OR "300.23"] AND

***Block 2*:** ["Physical activity" OR exercise OR "physical exercise" OR sport* OR fitness OR workout OR "physical training" OR "physical movement" OR movement OR "physical effort" OR athletic* OR "sport* involvement" OR "weight training" OR "strength training" OR "resistance training" OR "resistance exercise" OR "physical exertion" OR "recreational sport*" OR "amateur sport*" OR "professional sport*" OR "elite sport*" OR "competitive sport*" OR "individual sport*" OR "team sport*" OR "indoor sport*" OR "outdoor sport*" OR endurance OR "endurance sport*" OR "endurance exercise" OR gymnastic* OR "ball game*" OR "ball sport*" OR dance OR dancing OR dancer OR "perform* art*" OR ballet* OR ballroom OR walking OR swim* OR hike OR hiking] AND

***Block 3*:** [Treatment* OR intervention* OR RCT OR "randomized trial" OR "randomized controlled trial" OR "randomized-controlled trial" OR "randomised trial" OR "randomised controlled trial" OR "randomised-controlled trial" OR "clinical trial" OR "clinical study" OR "group comparison" OR "therap* evaluation" OR longitudinal OR therapy OR evaluation]
