# Supplementary material for: Physical Activity as a Treatment for Social Anxiety in Clinical and Non-clinical Populations: A Systematic Review and Three Meta-Analyses for Different Study Designs
Source: Front Hum Neurosci. 2021 Jun 11;15:653108. doi: 10.3389/fnhum.2021.653108 (PMC8230570; doi:10.3389/fnhum.2021.653108)
Supplement: Supplementary file 2 [file Data_Sheet_2.docx]

**Supplementary Material S2: PICOS Table**

***Abbreviations:***

BMI: Body-mass index

CBT: Cognitive-behavioral therapy

CL: Clinical

FNE: Fear of negative evaluation

GAD: Generalized anxiety disorder

MBSR: Mindfulness-based stress reduction

MDD: Major depressive disorder

NA: Not applicable

NCL: Non-clinical

PA: Physical activity

PEAT: Peer-enhanced adventure therapy

PPH: Perceived physical health

RCT: Randomized-controlled trial

SA: Social anxiety

SAD: Social-anxiety disorder

SPA: Social-physique anxiety

SPAS: Social-physique anxiety-state

SPE: Self-presentation efficacy

| **Study** | **Participants (P)** | | **Interventions (I)** | **Comparisons (C)** | **Outcomes (O)** | **Study design (S)** |
| --- | --- | --- | --- | --- | --- | --- |
| Ashdown-Franks et al. (2017) | Young adults, NCL | | NA | NA | Symptom reduction in anxiety subtypes in young adulthood; associations between number of years playing team or individual sport with anxiety subtype | Cross-sectional |
| Atalay et al. (2008) | Young adults, NCL | | NA | NA | The critical roles of exercising and body-image satisfaction on SPA and SA | Cross-sectional |
| Benau et al. (2020) | Young adults, NCL | | NA | NA | The interaction between gender and teammate interdependence of non-athletes, individual athletes, and team athletes on established psychosocial risk factors for disordered eating, including social phobia, alexithymia, and emotion regulation | Cross-sectional |
| Brière et al. (2018) | Adolescents, NCL | | NA | NA | Psychological difficulties (depressive symptoms, SA, loneliness) and differences in sport and personal characteristics | Longitudinal |
| Dimech et al. (2010) | Children, NCL | | NA | NA | The role of sport as a mediating variable in the onset or development of SA symptoms | Cross-sectional |
| Dimech et al. (2011) | Children, NCL | | NA | NA | The role of sport as a mediating variable in the onset or development of SA symptoms | Longitudinal |
| Dong et al. (2018) | Adolescents, NCL | | NA | NA | Internet addiction and its associated factors (gender, being the only child, father-adolescent relationship, annual family income, academic performance, physical exercise, self-esteem, and SA) | Cross-sectional |
| Goodwin et al., (2003) | Adolescents and adults, NCL | | NA | NA | Associations between PA, social network, and high levels of SA, as well as the strength of these associations in relation to an unhealthy lifestyle and a small social networks | Cross-sectional |
| Hartmann et al. (2010) | Children, NCL | | Daily physical education class in school, aerobic exercise, PA homework, encouragement of activities during breaks over one academic year | Usual school curriculum including three physical education lessons per week (45 min. each) | The association between FNE with PA; relationship between FNE and PPH and/or BMI | RCT |
| Have et al. (2011) | Adults, NCL | | NA | NA | The role of sport and sport mode (team vs. individual) as a mediating variable in the onset or development of SA symptoms | Longitudinal |
| Herring et al. (2014) | Young adults, CL | | NA | NA | Associations between self-reported PA and symptoms of SAD, generalized anxiety disorder, and obsessive–compulsive disorder, through the direct association with physical self-concept and self-esteem and the potential influence of BMI as a confounder of the relationship between PA and physical self-concept | Cross-sectional |
| Jamal et al. (2012) | Adults, CL | | NA | NA | Effect of smoking and nicotine dependency on the severity and course of depressive and anxiety symptoms in psychiatric people. | Longitudinal |
| Jazaieri et al. (2012) | Adults, CL | | Two weekly individual aerobic exercise sessions (at moderate intensity), and one group aerobic esxercise session over two months | Weekly MBSR sessions over two months | Social anxiety (SAD-related clinical symptoms) | RCT |
| Jelalian et al. (2011) | Adolescents, NCL | | Weekly CBT + PA (60 min. aerobic exercise) over 16 weeks | CBT + PEAT | Associations between change in BMI and psychosocial variables in adolescent perceptions of peer rejection and SA, as well as improvements in social functioning | RCT |
| Kim et al. (2020 | Adolescents, NCL | | NA | NA | Association between passive and active forms of screen time and adolescent major depressive episode and anxiety disorders (social phobia, generalized anxiety disorder, and specific phobia) | Cross-sectional |
| Koç et al. (2018) | Adolescents, NCL | | NA | NA | SA level and communication skills in terms of different variables (PA, sport type (individual vs. team sport), educational status of parents, PA of family members) | Cross-sectional |
| Lamarche et al. (2009) | Young adults, NCL | | 7 times step-aerobic class (7 min. warm-up + 20 min. step aerobics + 7 min. cool-down) | Part of the participants completed the class in front of a mirror, while the other participants completed the class without a mirror. [*This was not considered as control condition for the systematic review and meta-analysis*] | The influence of mirrors on self-presentation efficacy and state SA in a group exercise class | Longitudinal, intervention |
| Lamarche et al. (2010) | Young adults, NCL | | One time exercise ball-class designed for beginners (30 min.) | NA | The impact of the exercise leader’s gender on the self-presentational concerns of women in a live exercise class. SPE, state SA and SPAS, as well as perceived exertion | Longitudinal, intervention |
| Lőkös et al. (2013) | Children, NCL | | Swim training (twice a week for 60 min./session) or complex sports therapy (twice a week swim training (60 min./session) and once a week indoor and outdoor sports) over 18 months | Two matched (sex, age, and BMI) control groups, that did not receive any type of active intervention | Anxiety as a stable personality trait and state anxiety in relation to a present context; SA in children (SA-distress in new situations; FNE, SA-distress in general); test anxiety | RCT |
| Luna et al. (2019) | Adolescents, NCL | | 6 weeks physical sports program (2-3 sessions/week, 55 min. each session) | A didactic unit of traditional collective sports with a conventional teaching style, which aimed to improve students’ technical motor skills, 6 weeks, 2 sessions/week (12 x 55 min.) | The impact of a physical-sport education pilot programme on adolescents’ subjective well-being (health-related quality of life, positive affect, negative affect, trait emotional intelligence, as well as SA) | Longitudinal, intervention |
| Mamen et al. (2011) | Young adults and adults, CL (all had a diagnosis of substance abuse/dependence and co-morbid psychiatric disorders) | | 11 months individual training (jogging, cycling, cross-country skiing, and swimming, aerobic exercise, mountain hiking, and ball games, competitions for recreational athletes, both individually and in teams) | NA | The relationship between improvements in physical fitness and mental health (mental distress, depression, anxiety, SA, alcohol, and drug abuse) | Longitudinal, intervention |
| Özşahin et al. (2018) | Adults, NCL | | NA | NA | Associations between SAD and obesity as well as risk factors for SAD | Cross-sectional |
| Ren et al., (2020) | Adolescents, NCL | | NA | NA | Influence of physical exercise on SA of left-behind children in rural areas, as well as the role of perceived social support | Cross-sectional |
| Rocha et al. (2014) | Young adults, NCL | | NA | NA | Influence of regular PA on musical performance anxiety | Cross-sectional |
| Shin et al. (2018) | Adults, CL | 8 weeks, 3 times/week for 70 min., circuit-training program | | Different feedback groups within the circuit-training program (positive feedback, negative feedback, mixed feedback, and no feedback) | Effects of circuit training on mental health (SA, anxiety, positive emotion) and physical health (body composition, physical fitness) of workers with SAD. | Longitudinal, intervention |
| Üstün et al. (2019) | Adolescents, NCL | NA | | NA | Participants' perceived SA levels according to their sports branch (individual or team sports), the weekly duration/day of doing sports, the aim for doing sports as well as gender | Cross-sectional |
| Yu et al. (2020) | Children, NCL | School-based nutrition education and PA over 8 months: daily on school days (20 min. jogging + one extra 40 min. gym class (rope skipping, badminton, and 200-m relay race) + PA educational class 4 x 60 min.) + PA educational class every second month | | Usual practice with no extra intervention in school; the parents of the children in the CG received the results of anthropometric and blood test results and simple suggestions about healthy lifestyles to reduce their children’s body weight) | Effects of activity intervention on mental health outcomes (well-being, depressive symptoms, and SA), obesity measures (BMI, fasting blood glucose, and lipids) | RCT |
| Zink et al. (2019) | Adolescents, NCL | NA | | NA | Association between screen-based sedentary behaviors and emotional disorders (MDD, GAD, Panic Disorder, and SAD) | Longitudinal |
